# Factors associated with persons with disability employment in India: a cross-sectional study

**DOI:** 10.1186/s12889-016-3713-6

**Published:** 2016-10-07

**Authors:** Ramya Naraharisetti, Marcia C. Castro

**Affiliations:** Department of Global Health and Population, Harvard T.H. Chan School of Public Health, 665 Huntington Avenue, Bldg I, Room 1113, Boston, MA 02115 USA

**Keywords:** Disability, India, Employment, Persons with Disability

## Abstract

**Background:**

Over twenty million persons with disability in India are increasingly being offered poverty alleviation strategies, including employment programs. This study employs a spatial analytic approach to identify correlates of employment among persons with disability in India, considering sight, speech, hearing, movement, and mental disabilities.

**Methods:**

Based on 2001 Census data, this study utilizes linear regression and spatial autoregressive models to identify factors associated with the proportion employed among persons with disability at the district level. Models stratified by rural and urban areas were also considered.

**Results:**

Spatial autoregressive models revealed that different factors contribute to employment of persons with disability in rural and urban areas. In rural areas, having mental disability decreased the likelihood of employment, while being female and having movement, or sight impairment (compared to other disabilities) increased the likelihood of employment. In urban areas, being female and illiterate decreased the likelihood of employment but having sight, mental and movement impairment (compared to other disabilities) increased the likelihood of employment.

**Conclusions:**

Poverty alleviation programs designed for persons with disability in India should account for differences in employment by disability types and should be spatially targeted. Since persons with disability in rural and urban areas have different factors contributing to their employment, it is vital that government and service-planning organizations account for these differences when creating programs aimed at livelihood development.

## Background

According to the 2001 Indian Census, there were 21.9 million people (2.1 % of the population) living with disability, the majority located in rural areas (75 %) and most unemployed (65.5 %) [1]. Understanding the differential employment of persons with disability (PwD) is especially relevant since in the last two decades the national government has adopted progressive disability law.

Indian disability legislation dates as far back as the 1987 Mental Health Act [2], followed by the 1992 Rehabilitation Council of India Act [3], which supported the growth of human resources within the disability rehabilitation sector. India was the first nation in South Asia to sign the Proclamation on the Full Participation and Equality of People with Disabilities in the Asian and Pacific [4]. This resulted in the 1995 Persons with Disabilities (Equal Opportunities, Protection of Rights and Full Participation) Act [5]. The Act was known to be one of most comprehensive pieces of legislation pertaining to persons with disabilities in the region. Specific to employment, it had provisions on non-discrimination in the built environment and in government employment. It gave statutory recognition to an employment reservation policy of 3 % in government and public education institutions. Specifically, a 1 % reservation is required for three disability categories combined: hearing, vision and locomotor. Further, an unemployment allowance exists for those registered with the Special Employment Exchange program (a national employment service) for more than two years without securing employment.

Since poverty is the greatest challenge before planners in India, and the incidence of disabilities is very high in rural (75 % of total) and poor families, the 1995 Act has mandated the government to include PwD in all its mainstream poverty alleviation programs. The Act states that, at all levels, the government shall reserve not less than 3 % of all funds in poverty alleviation programs for the benefit of PwD. Nevertheless, the 1995 Act has shortcomings, such as vague terminology, and gaps regarding the implementation, monitoring and evaluation of programs. Further, the Act states that reforms should only be adopted “within the limits of the state’s capacity”. There are no mechanisms empowering any authority or court to impose fines or levies in the case of the breach of the provisions relating to training and employment [6]. Overall, the Act has failed to improve the lives of persons with disabilities and protect their human rights [6].

In 2007 India signed and ratified the Convention on the Rights of Persons with Disability (CRPD), absent of the Optional Protocol (which provides an internationally recognized mechanism to ensure that rights are realized through systematic reporting and evaluation of countries by established international committees). Although it was enacted in 2008, comprehensive reforms are yet to take place [6]. Given the large discrepancies between the approach of the Indian government and the CRPD, there are debates about whether the previous disability legislation should be reformed or a new act should be created.

Although the legislation focused attention on disability, there has been a noted lag in implementation of services and programming for PwD [7–9]. This is especially true in rural areas, since the relatively few public rehabilitation services are mostly located in urban centers [10]. As governmental and non-governmental agencies begin to address this gap, it remains unclear what factors contribute the most to PwD employment, and whether their employment experiences differ geographically across India.

Considering labor market supply, PwD experience barriers of accessibility to and ability at the workplace. Productivity is largely dependent upon the characteristics of the type of disability and the requirements of the job. For example, a person with hearing impairment may find it difficult to do telecommunication work, but excel in mathematically-based accounting work. Overall, according to labor market theory, a higher reservation wage and a lower market wage make a PwD less likely to be employed than a person without disability [11].

Discrimination can play a role when PwD with equal productivity to those with no disability have unequal opportunities. There has been a dynamic shift from the *medical* model of disability to the *social* model [12]. The social model of disability draws a clear distinction between impairments and disability by clarifying that the degree of disability is a function of the societal barriers that fail to accommodate difference. A bio-pyschosocial definition of disability is reflected in the Preamble of the Convention on the Rights of Persons with Disability’s (CRPD) definition of disability as well [13]. Despite this conceptual shift, the official discourse continues to perceive disability as purely a medical condition, framing the individual on his/her own without engaging with the wider social and physical context [14]. Scholars argue that this has led to a “top-down” approach where blanket policies are applied to all PwD with a disregard for heterogeneity in experiences.

The International Labor Organization (ILO) has outlined two categories of factors which affect full participation of PwD in the Indian labor market [15]: environmental and social. Physical environment and public facilities and utilities have not been developed or designed with the requirements of each category of disability in mind and nearly all mainstream training programs and work sites exclude disability groups due to these barriers. For example there are over 100 regional sign languages, but there is no acceptable national sign language for use by all people with speech/hearing impairment during vocational training sessions [16]. Social barriers comprise a critical impediment in the process of full participation. Baldwin and Johnson [17] explain that employment discrimination can occur due to prejudice, differential information about the average productivity of persons with and without disabilities, or the exploitation of workers by employers [13, 17].

In India, research on disability has been limited by the availability of data. For example, the National Sample Survey has never collected data on employment across disability status. The 2001 Indian Census included multiple questions on disability, and also collected data on literacy, sex, and employment status. No detailed information on amount of education and level of income were included. This paper takes advantage of this information available on the Census to investigate the correlates of the proportion of PwD employed in India, accounting for geographic variation at the state and residential (rural and urban) levels. Results have direct implications for the planning of interventions targeted to PwD. To the best of our knowledge, this is the first study to investigate the differential determinants of employment among PwD in rural compared to urban areas in India, accounting for potential spatial effects.

## Methods

### Disability definition

The most common definition and classification of disability within the Indian government was determined with the enactment of the 1995 Act, and states that a person is considered to have a disability if they suffer ‘from not less than 40 % of any disability as certified by a medical authority’ [5]. Disability is considered to be blindness, low vision, leprosy-cured, hearing impairment, locomotor, mental retardation, or mental illness. In 1999, the National Welfare of Persons with Autism, Cerebral Pulsy, Mental Retardation and Multiple Disability Act, added two classes: people with autism and people with multiple disabilities [18]. The 2001 Indian Census states that “defining and measuring disability is a complex issue and it is not easy to communicate these concepts during the census process, in which only a limited amount of questioning time is possible with a household for obtaining detailed information on every individual”. The Census therefore used its own version of disability types, classified into five categories: (i) sight (ii) speech (iii) hearing (iv) movement and (v) mental [1]. This definition has been accepted by the government, both administratively and legally, and is thus used in this paper.

### Data

The data source for this study is the 2001 Population Census, which included multiple questions on disability. Each person was asked if he/she had a physical or mental disability according to five categories: speech, sight, hearing, mental (mental), or movement (physical) [19]. If a person has two or more types of disabilities only one was recorded, and it was left to the respondent to decide which one they wanted to be classified into as the most dominant. This was a choice made by the Government of India’s Census Office at the time. Persons with temporary mental or locomotor inability (due to acute medical conditions) on the date of enumeration were not considered as disabled.

The dependent variable in this research is employment and it is defined as those that participated in “work” according for the Indian Census. The Census defines work as “participation in any economically productive activity with or without compensation, wages or profit. Such participation may be physical and/or mental in nature. Work involves not only actual work but also includes effective supervision and direction of work. It even includes part time help or unpaid work on farm, family enterprise or in any other economic activity”. There are several categories of “work” used by the Census including main worker, marginal worker, cultivator, agricultural laborers, household industry workers and other workers [20]. According to the Census 2001 metadata, ‘the reference period for determining a person as worker and non-worker is one year preceding the date of enumeration”.

The disability data were detailed by district, which is the first-level administrative unit within an Indian state. There are 890 districts within the 28 states and seven union territories of India. Of these, 47 are island districts (such as the Andaman and Nicobar Islands, which have no neighboring districts and therefore are not suitable for spatial analytical methods). Of the remaining 843 districts, 250 have no inhabitants. Percentages were calculated considering only the 593 remaining districts with reported inhabitants in the Census. Each district can have both rural and urban areas, and a small number are considered as exclusively rural or urban; thus the denominator for urban and rural percentages varied. In the urban/rural analysis rural and urban percentages were calculated for each variable. We started with a dataset that was stratified by rural and urban PwD from the Indian Census, so the districts did not have to classify as urban and rural. Table 2 reports the total 843 because all districts are used regardless of their inhabitants in the spatial model, since it will exclude those districts automatically.

The disability data were spatially joined to the 2001 Census geographic dataset (retrieved from Harvard GeoSpatial Library, Cambridge, Massachusetts). The data were projected using Kalianpur 1975 India Zone IIb, which is a spatial adjustment to view a specific part of the globe in a flat way. Data joining and projection were done in ArcGIS 10 (Environmental System Research Institute, Redlands, California). The regression was completed in GeoDa^TM^. The research was ethically approved by the Harvard School of Public Health’s Department of Global Health and Population as a part of the fulfillment for a Master’s of Science and it was determined that International Review Board submission was not necessary due to the analysis of secondary data from the Indian Census which is publically available.

The main variable of interest is the proportion of employed PwD in a district. The variable was calculated as a rate: the total number of employed persons with disability in a district as the numerator and the total number of persons with disability in the denominator. Employment is defined as six types of “workers”: main workers, marginal workers, cultivators, agricultural labourers, household industry workers and other workers. Other relevant variables, all at the district-level included: (i) proportion of female PwD; (ii) proportion of PwD who are literate; (iii) proportion of PwD by disability type; and (iv) proportion of PwD living in urban areas. These variables were calculated with all ages of PwD as the denominator, except for the literacy variable. Age restrictions were not included in the data because this information was not available. Therefore, this analysis should only be interpreted as the proportion of employed persons with disability of total persons with disability in a district. There is potential confounding due to variation in age distributions between districts but this is likely small. Population density (total number of persons with and without disability) was also considered and not included because persons with disability are looked as a separate population in this analysis. Further, data was available for only those that lived in urban and those that lived in rural areas (as separate data sets). This was used in a stratified analysis of rural and urban populations to identify any patterns in characteristics that predicted employment.

In the 2001 Census, literacy was defined as the ability to read in the local language. In this research it is used as a crude estimate to determine whether someone has at least a few years of schooling. Although the ideal variable would be years of education, this information was not collected for PwD in the 2001 Census. We would expect this variable to predict employment because of the intimate link between education and employment that is experienced throughout India, especially among PwD [8, 21]. Therefore we may expect literacy to positively predict employment.

### Analytical approach

Linear regression models were used to assess potential determinants of the proportion of PwD employed at the district-level. Linear regression has several assumptions, which were assessed. Potential covariates were chosen based on three criteria: (i) evidence from the literature regarding common drivers of employment and those specific to PwD, in India and in other countries; (ii) special attention to variables that can contribute to the formulation of state and local policy; and (iii) the availability of data in the 2001 Indian Census at the district-level.

Three model formulations were considered. The first (labeled as Model 1) included (i) proportion of female PwD in a district; (ii) proportion of illiterate PwD in a district; (iii) proportion of PwD by disability type in a district, considering four categories: mental, movement, sight, and speech/hearing (combining speech and hearing in one category is plausible because they can be considered communication disorders that generally (but not always) occur together [22]; and (iv) proportion of PwD living in urban areas in a district. The second model (labeled as Model 2) included all variables from Model 1 and added state fixed effects in order to account for potential correlation between the proportion of PwD employed and state characteristics.

Since previous studies have shown that employment for PwD is more difficult in rural areas [11, 23], compared to urban areas, we stratified the analysis by area of residence. We considered the model formulation with greatest explanatory power (as defined by the R^2^ observed in Models 1 and 2) and ran two additional models, one for urban and another for rural PwD. Further stratification could have been pursued based on variable distribution. The purpose of the model was not to account for the differences in distribution of disability characteristics. It was a cross sectional look at how the distribution of these characteristics influenced employment.

Lastly, we considered that the proportion of PwD employed could vary spatially [13, 24, 25], and in this case the presence of spatial autocorrelation would violate basic assumptions of linear models [26]. Thus, we tested the residuals of each model for the presence of spatial autocorrelation using the global Moran’s I indicator. If the test was significant, we used spatial autoregressive models, and included spatial lag terms based on diagnostics provided by Langrage Multiplier tests [26]. Model goodness-of-fit was assessed by comparing the likelihood ratio and the Bruesch-Pagan test of each model. The Breusch-Pagan test is used to compare the standardized square of the OLS residuals regressed against the square of the original coefficients to determine the presence of heteroskedasticity in the error terms. All regression models were run in GeoDA, an open-source spatial analysis software.

## Results

### Disability in India

In 2001 there were 22 million people living with a disability in India, corresponding to 2.1 % of the population or 21 disability cases per 1000 [27]. Table 1 presents descriptive statistics for PwD in India in 2001 by area of residency. The most common disability type at the district-level was sight (47.5 %), followed by movement (27.7 %) and mental (10.1 %). On average, 46.6 % of PwD in a district were literate (compared with 64.8 % literacy among the general population in 2001), with a mean of 57 % literacy among males and 35 % literacy among females [28]. These statistics varied in rural compared to urban areas, particularly regarding illiteracy, employment, hearing disability, and mental disability.

**Table 1:** Characteristics of PwD (% in the district) in India detailed by area of residence - 2001

Characteristics	Total (*n* = 593 districts)	Rural (*n* = 583 districts)	Urban (*n* = 584 districts)
Seeing	47.5 % (0.103)	46.7 % (0.104)	48.92 % (0.129)
Hearing	6.8 % (0.034)	7.16 % (0.035)	4.92 % (0.023)
Speech	7.8 % (0.026)	7.89 % (0.027)	7.54 % (0.024)
Movement	27.7 % (0.081)	28.5 % (0.082)	27.15 % (0.093)
Mental	10.1 % (0.033)	9.76 % (0.032)	11.45 % (0.039)
Female	42.3 % (0.039)	42.5 % (0.039)	41.2 % (0.047)
Illiteracy	53.4 % (0.101)	55.94 % (0.097)	38.36 % (0.072)
Employment	36.0 % (0.072)	37.34 % (0.074)	29.43 % (0.073)

Standard deviations presented in parenthesis

Source: Author’s Calculation from 2001 Indian Census

There was great variability of characteristics within PwD who were employed. Of all employed PwD, 58.3 % were either cultivators or agricultural laborers, 4.6 % were household workers, and 37.3 % were classified as other (data not shown). This could explain the higher proportion of employed PwD in rural areas, possibly due to the greater availability of agricultural work. Employment among males and females was 44.8 % and 29.6 %, respectively (compared to an employment rate of 39.1 % among the general population) (data not shown) [28]. The difference in worker participation rates between males and females was larger in the urban areas when compared to rural, and the lowest worker participation rate was for urban females (29.4 %).

Adding state fixed effects to this model increased the R^2^ (from 42 to 76 %), and changed the magnitude and direction of some coefficients, indicating that state-specific characteristic play a role in levels of employment among PwD.

The final model was stratified by rural and urban and included state fixed effects (Table 2). No disability type was associated with fewer PwD employed in urban areas. While there would be a 0.455 percentage point decrease in PwD employed in urban areas for each additional unit of female PwD, in rural areas the association is the opposite, with a 0.629 percentage point increase in PwD employed for each additional unit of female PwD. This could be the result of more opportunities for agricultural employment among women in rural areas. In this stratified model, illiteracy is negatively associated with PwD employed, with 0.039 and 0.265 percentage point decrease in PwD employed in rural and urban areas, respectively, for each additional unit of illiterate persons.

**Table 2:** Stratified regression models on the proportion of employed PwD by District^c^ in India, 2001

	Model 1: Stratified		Model 2: Spatial Error Model	
Variable	Rural	Urban	Rural	Urban
Constant	0.0090 (0.0211)	0.0230^a^ (0.0101)	−0.0181 (0.0165)	−0.0114 (0.0084)
Movement	−0.0285 (0.0356)	0.1649^b^ (0.0315)	0.3161^b^ (0.0412)	0.4776^b^ (0.0340)
Mental	−0.7955^b^ (0.1000)	0.2293 (0.0646)	−0.4046^b^ (0.1030)	0.3778^b^ (0.0620)
Sight	0.2012^b^ (0.0352)	0.4575 (0.0322)	0.3899^b^ (0.0350)	0.6200^b^ (0.0308)
Female	0.629^b^ (0.0547)	−0.4551^b^ (0.0428)	0.2529^b^ (0.0580)	−0.5614^b^ (0.0399)
Illiterate	−0.0387 (0.0296)	−0.2651^b^ (0.0273)	0.0350 (0.0297)	−0.1225^b^ (0.0271)
Spatial Error			Yes	Yes
State Fixed Effects (No. Categories)	Yes(21)	Yes (21)	Yes (21)	Yes (21)
R-Squared	0.4753	0.6695	0.6967	0.7800
No. Obs.	843	843	843	843
Global Moran’s I	19.0492^b^	14.6926^b^		
Lagrange Multiplier (lag)	96.1904^b^	64.9307^b^		
Robust LM (lag)	13.0312^b^	13.8244^b^		
Lagrange Multiplier (error)	321.4412^b^	136.9104^b^		
Robust LM (error)	136.9104^b^	239.0752^b^		
Breusch Pagan-Test			323.7729^b^	392.6021^b^
Likelihood Ratio Test			350.1907^b^	252.7653^b^

Robust standard errors are reported in parentheses

^a^, ^b^ indicates significance at the 95 %, and 99 % level, respectively

^c^Total number of persons with disability in India in 2001 = 22 million persons or 2.1 % of the total population

This stratified model, however, had very significant spatial autocorrelation in the residuals (as indicated by the highly significant Moran’s I test), and thus the coefficients are likely to be biased. Based on the Robust Lagrange Multiplier test spatial autoregressive models considering a lag term on the residuals (labeled as Spatial Error Model in Table 2) were fit. They showed improvements in the R^2^ of both the urban and rural models, and differences in magnitude, direction, and significance of coefficients (as compared to the non-spatial stratified model). Controlling for spatial effects, no type of disability is associated with a decrease in the percentage point of PwD employed, with the exception of mental disability in rural areas. A unit increase in mental health related PwD was associated with a 0.404 percentage point decrease in PwD employed in rural areas, whereas a unit increase in PwD with mental health was associated with a 0.378 percentage point increase in PwD employed in urban areas. The negative association between females and PwD employment and illiteracy and PwD employment for urban areas observed in the first model persists after controlling for spatial effects. A unit increase in female PwD was associated with a 0.561 percentage point decrease in PwD employed in urban areas. A unit increase in illiterate PwD was associated with a 0.123 percentage point decrease in PwD employed in urban areas.

Results of the Breusch-Pagan test suggest that heteroskedasticity remains after introducing the spatial lag term. In addition, the Likelihood Ratio test of Spatial Lag Dependence is also significant. Therefore, although the introduction of the spatial lag term improved the model fit, it did not completely remove the spatial effects.

## Discussion

Our results showed that disability type, gender, and illiteracy were associated with PwD employment, and that the magnitude and direction of the association was not the same for urban and rural areas. Being a female and being illiterate results in less PwD employment in urban areas, while having mental disability results in fewer PwD employed in rural areas. Having movement and sight disability (holding all other variables constant) resulted in increased PwD employment in urban and rural areas. This is expected because most public programs target movement and sight disability [29]. The presence of spatial effects underscores the need to properly address local idiosyncrasies in policies and programs aimed to improve employment of PwD.

Regarding policy and programmatic issues, strategies implemented in urban areas need to address gender and illiteracy discrimination. While these results align with previous research [30–33], it is important to identify if there are fewer jobs available for female PwD and for illiterate PwD, if these people lack the proper training to undertake the job, or if they are qualified but do not have equal job opportunities. Moreover, it is crucial to assess the extent to which these issues vary across districts and states. The fact that in rural areas gender and illiteracy is not associated with fewer PwD employment could be a result of the prominence of agricultural labor [7, 11, 23, 34].

Current disability rights legislation for employment assumes homogeneity of experiences. Most efforts target PwD with vocational training and employment services through Federal mandates of “one size fits all” type of policy. There are although a few state-government initiated pilot programs which challenge this approach. For example, the state of Andhra Pradesh and the city of Pune have explored the utility of disability self-help groups that provide more autonomy to local leaders for rural development [7]. Self-help groups take the form of neighborhood-based collectives, which actively participate in problem solving and in the development of local programming. Self-help groups have been widely employed for women, but its application to PwD is less prevalent [35–39]. They mostly take the form of microcredit-based interventions that provide entrepreneurial opportunities. Their applicability beyond economic empowerment has although been the most salient feature. They allow for PwD communities to systematically organize around issues that are pertinent and pressing to them, allowing for programming to be more local and therefore more contextualized.

Regarding disability type, mental problems seem to be a major barrier for PwD employment in rural areas. This could be a result of lack of jobs suitable to persons with mental disabilities, of discrimination, or of lack of minimum training to conduct the job. This has important implications for targeted initiatives. The Rural Health Commission and the National Rural Employment Guarantee Act target persons in rural areas, without attention to specific disability types. For example, in the state of Andhra Pradesh, the Society for the Elimination of Rural Poverty has piloted microcredit schemes for PwD in rural areas, targeting all PwD, with no differential interventions for particular disability types. Also, a governmental agency based in south eastern India called the National Institute for the Mentally Handicapped has developed extensive vocational training for PwD, which has proven effective in increasing employability [40], but their scope is limited to urban areas. Another, non-governmental organization called Maharogi Sewa Samiti based in central India has developed a vocational training center for rural school drop outs and youth with disabilities. Although they target PwD in rural areas, all of the training occurs in the urban centers causing low retention rates. A similar initiative has been initiated by one governmental agency, the Society for the Elimination of Rural Poverty in Hyderabad. But they face similar issues with the context of an urban-based training approach.

This study has some limitations. Our data do not allow making conclusions about causality. Nevertheless our modeling approach and results shed light into factors associated with PwD employment, and make it clear that programmatic efforts designed to improve the wellbeing of PwD must account for disability type and spatial differences. In addition, we had a limited set of variables to include in our models. For example we would expect age and socioeconomic status to play a role in employment. Yet, the models had a high explanatory power, for both urban and rural areas. Another limitation is that the definition of disability varies across different surveys, and therefore caution is needed when attempting to establish comparisons between our results and others previously published [14]. Further, the decision of the Census of India to not include multiple disabilities as a category has implications for the interpretation of results. Mainly, caution should be used when extending these results to the small percentage of people with multiple disabilities. A recent article on disability estimates from the 2001 Census and the 2002 58th round of the National Sample Survey (NSS) concluded that “prevalence estimates in the census and the NSS are clearly not comparable… and it is unsure what aspects of disability are captured by the census and NSS current disability definitions” [11]. Lastly, the analysis was limited to district-level number of PwD; thus, due to the lack of individual-level data, the model predicts the likelihood of having lower or higher proportion of (percentage point increase or decrease in) PwD employed within a district, based on the type of disability and other variables.

## Conclusion

Persons with disability have different employment experiences depending on their disability type, literacy, gender, and on whether they live in rural or urban areas. Although India has achieved significant progress regarding disability legislation, and has recently made an effort to implement poverty alleviation strategies targeting PwD, failure to account for these differences may hinder the benefits of the efforts.

Policies implemented at the national level without considering local idiosyncrasies are not likely to result in equitable improvements for the livelihood of PwD. There is a need to better understand the barriers to PwD employment, and how those barriers may vary by disability type across different districts in India. Such knowledge would provide much needed evidence that could be translated in more effective local policies. The results presented here are a first step towards building this knowledge by unveiling significant differences across urban and rural areas, type of disability and gender.

## Abbreviations

CRPD: Convention on the rights of persons with disabilities
ILO: International Labor Organization
NSS: National Sample Survey
PwD: Persons with disability
